# Green Development Performance Evaluation Based on Dual Perspectives of Level and Efficiency: A Case Study of the Yangtze River Economic Belt, China

**DOI:** 10.3390/ijerph19159306

**Published:** 2022-07-29

**Authors:** Rui Zhang, Yong Ma, Jie Ren

**Affiliations:** 1 School of Business, Hubei University, Wuhan 430062, China; 202011112010088@stu.hubu.edu.cn; 2 Center for Tourism Development and Management Studies of Hubei, Wuhan 430062, China; mayong2@hubu.edu.cn; 3 School of Tourism, Hubei University, Wuhan 430062, China; 4 College of Business Administration, Zhongnan University of Economics and Law, Wuhan 430073, China

**Keywords:** green development level, green development efficiency, entropy weight TOPSIS, super-EBM model, influencing factors, Yangtze River Economic Belt

## Abstract

In the context of continuing to promote the construction of an ecological civilization, it is of great significance to explore green development performance. However, most of the literature is based on a single perspective of level or efficiency, lacking a comprehensive examination of both. It is not scientific to explore how to promote green development only from a single perspective, which may be a new advancement by breaking the conventional thinking focusing only on level or efficiency. On this basis, we first established evaluation index systems of green development performance based on a theoretical framework. Furthermore, green development performance was measured with the entropy weight technique for order preference by similarity to ideal solution (TOPSIS) and super-EBM models, and finally, we analyzed the spatial and temporal evolution patterns of green development performance using the ESDA method and examined its influencing factors with a geographic detector (GD) and econometric models. The main results were as follows: (1) The trend of the green development level in the Yangtze River Economic Belt from 2004 to 2017 had an inverted “N” shape, while the overall average green development efficiency continuously increased. (2) In terms of spatial and temporal patterns, both the green development level and green development efficiency showed “high in the east and low in the west” spatial divergence characteristics. In terms of the spatial and temporal evolution pattern of the green development level, the L-L clusters were mainly distributed in the western region. However, for green development efficiency, the L-L clusters were mostly distributed around the H-H clusters. (3) The results of the influencing factor analysis indicated that industrial structure and people’s welfare are still important factors of the green development level. The improvement of green development efficiency was mainly driven by economic development, and the inhibiting effect of energy consumption is significant. In addition, the effect of opening up has not yet changed from a “pollution paradise” to a “pollution halo”.

## 1. Introduction

Production elements are increasingly accumulated as a result of economic reform and infrastructure improvements, and China’s economy has achieved fast growth and transformed the pattern [1]. However, the resulting resource and environmental limits have worsened, and the contradiction between industrial society and the natural environment has become a major stumbling block to long-term economic and social progress [2]. Since the first environmental conference held in Stockholm in 1972, China has been paying more attention to environmental issues. Sustainable development, green development, energy conservation, and emission reduction have been incorporated into strategic systems as major policy objectives. As a result, the traditional crude development model with high energy consumption and low efficiency is gradually transformed into an intensive and efficient green development model, which means that relevant initiatives led by the green development concept will become an effective way to boost China’s sustainable economic and social development. However, according to the Global Environmental Performance Index (EPI) 2020 Report [3], China’s EPI score is only 37.3, ranking 120th out of 180 countries and regions, which indicates that China has achieved greater success in green transformation, but it is still inadequate compared to other countries. Therefore, how to decouple regional economic development from environmental pollution and promote the synergistic development of the economy, society, and eco-environment are still important issues [4]. As an important strategic region in China [5,6], the Yangtze River Economic Belt has become one of the leading demonstration areas for the green transformation of China’s economic development due to its resource endowment and high industrial agglomeration. In the context of China’s comprehensive promotion of green development, taking the Yangtze River Economic Belt as the research area to explore the temporal and spatial evolution characteristics and influencing factors of its green development performance is of great practical significance for China to further promote green development. Specifically, we mainly focus on the following questions: (1) How effective is the Yangtze River Economic Belt in promoting green development? (2) What are the temporal and spatial evolution characteristics of green development performance? (3) What factors drive or restrict green development?

To answer the above questions, based on data availability and established studies [7,8,9], we took 107 prefecture-level cities in the Yangtze River Economic Belt as research objects, systematically composed the theoretical framework of green development; constructed green development performance evaluation index systems based on two aspects, namely level and efficiency; and measured the green development performance from 2004 to 2017 using entropy weight TOPSIS and the super-efficiency EBM model. The spatial and temporal evolution characteristics of urban green development performance were analyzed with ESDA methods, and the mechanism of the influencing factors was explored with GD and econometric models. We explored the law of the spatial and temporal evolution of green development performance, clarified the mechanism, and provide a reference basis for the coordinated development of the three subsystems of economy, society, and ecological environment in the Yangtze River Economic Belt. The potential contributions of this study are as follows: (1) in terms of research perspective, the spatial and temporal evolutionary characteristics of green development performance in the Yangtze River Economic Belt were analyzed in two dimensions, namely level and efficiency, and the similarities and differences were discussed; (2) we further expanded the literature in the field of green development research at the prefecture-level city scale by taking cities as the basic unit; and (3) the mechanism of the influencing factors on green development performance was initially explored based on geographic detector (GD) and econometric models.

The structure of this paper is as follows: Section 2 provides the literature review. Section 3 constructs the evaluation index system for the green development level. Section 4 describes the materials and methods. Section 5 analyzes the empirical results. Section 6 provides the conclusion and discussion.

## 2. Literature Review

Green development, as an important yardstick to measure the coordinated development of economic, social, and ecological environment systems, has long attracted the attention of the government, and much research has been conducted by academics. The literature on topics related to green development performance mainly focuses on the construction of index systems, measurements, and influencing factors.

The evaluation index system is mainly divided into two categories: the green development level evaluation index system and the green development efficiency evaluation index system. Specifically, there are differences among similar index systems due to differences in research perspectives, research units, and research scales. (1) Green development level: International institutions and researchers have constructed corresponding indicator systems according to the different scales of the research objects, mainly focusing on economic, societal, and environmental aspects [7,8,10,11,12,13,14,15]. (2) Green development efficiency: Based on the input-output model, scholars have constructed green development efficiency evaluation index systems, including economic input, capital input, resource input, desirable output, and undesired output [16,17,18,19,20,21].

In terms of the measurement of green development performance, the green development level is mostly measured by entropy weight, entropy weight TOPSIS, coefficient of variation, and principal component analysis [16,17,22,23], while the measurement methods of green development efficiency mainly include the single-indicator method, indicator system method, stochastic frontier analysis method, and data envelopment analysis method [24,25,26,27,28].

Regarding the exploration of the influencing factors of green development, researchers often use the obstacle degree model, GD, Tobit regression, bootstrap truncation regression, spatial autoregression, and other methods to explore economic development, industrial structure, technological innovation, and urbanization at the national, provincial, or enterprise scale [29,30,31,32,33,34,35,36].

Summarizing the existing studies, it can be found that (1) the research content mainly focuses on one of the aspects of green development level or green development efficiency, which makes it difficult to reflect the real situation of green development in a comprehensive way; (2) in terms of evaluation index system construction, there is still space for expansion. For example, the literature on the evaluation index system of green development level lacks the consideration of people’s welfare. (3) In terms of research scales, most of the previous studies are based on national or provincial scales, and there is still space to expand the research on the fine-grained examination of urban scale.

## 3. Evaluation Index System for Green Development Performance

Green development is a complex system involving economic, social, resource, and environmental factors, and its theoretical premise is the symbiosis of economic, social, and ecological environment systems. Its core connotation is to enhance economic vitality, promote social welfare and resource wealth as the goal, and promote the decoupling of economic and social development and resource and environmental consumption [23,37,38].

**Figure 1:**
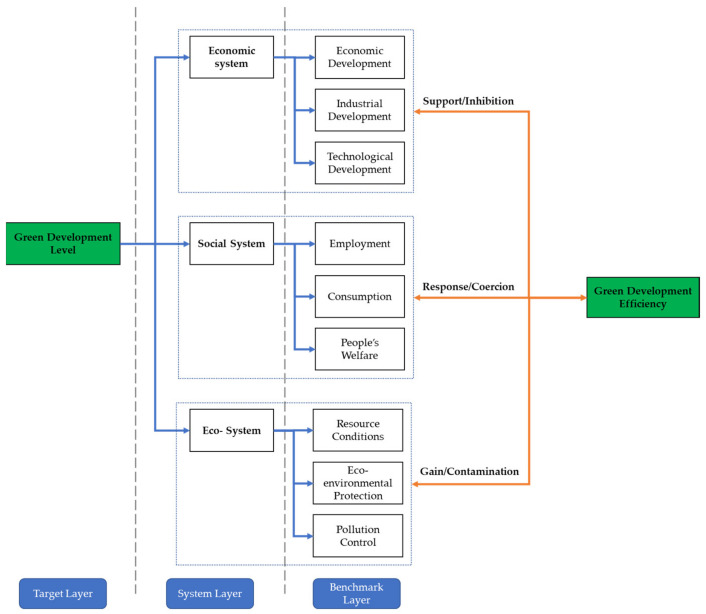
The theoretical framework of green development theory.

Currently, the concept of green development is rather vaguely defined and mostly focuses on a certain system of economy, society, or ecological environment, such as green economy, green production, and green innovation. We attempted to combine previous studies to sort out and define the theoretical framework of the green development level by integrating the three systems of economy, society, and ecological environment (Figure 1) [39,40,41]. The theoretical framework was formed by the compound of the target layer, system layer, and benchmark layer. (1) The economic subsystem consists of economic development, industrial development, and technological development, which supports green development. (2) The social subsystem contains employment, consumption, and people’s welfare. In the context of green development, more emphasis should be placed on social welfare and people’s welfare. (3) The interactive coupling of resource conditions, ecological environmental protection, and pollution control in the ecosystem subsystem forms the basic guarantee of green development. In summary, the system layer was formed by the integrated action of all elements in the benchmark layer, and furthermore, the coupling and symbiosis of all subsystems in the system layer finally form the target layer. Therefore, green development is a sustainable development concept that integrates the three subsystems of economy, society, and ecology and is based on benchmarks such as development capacity, social welfare, and ecological resources.

Furthermore, the internal mechanism of green development efficiency, which consists of economic, social, and ecological systems, was analyzed. The economic system is the basis of green development efficiency improvement; yet, it may also cause a constraining effect due to the crude economic growth model. The social system embodies the basic goal of green development at the social level, while it may also become a constraint on development. The eco-environmental system is the resource base for green development efficiency enhancement, which can play a role in improving efficiency but may also produce self-consumption in the process of efficiency enhancement.

Based on the above conceptual connotation and theoretical basis, we followed the three principles of systematicity, scientific analysis, and data availability and constructed evaluation index systems of green development performance, aiming to quantitatively assess it.

### 3.1. Evaluation Index System for Green Development Level

The green development level integrates the sustainability of the three subsystems of economy, society, and ecological environment. Based on the above analysis and existing studies [7,8,9,23,37,38,39,40,41,42,43], we constructed an evaluation index system of the green development level of cities in the Yangtze River Economic Belt based on data availability, with the green development level as the target layer and growth quality, industrial development, resource utilization, environmental carrying capacity, environmental governance, and green life as the guideline layer, as shown in Table 1.

Growth quality, as a continuous booster to maintain the green development of the Yangtze River Economic Belt, is an important economic foundation for development [37,38,39]. As a globally influential riverine economic zone, the Yangtze River Economic Belt has a high economic density, and the economic development level plays a pivotal role in green development.

Industry development is the economic support to ensure the green development of the Yangtze River Economic Belt [37,40]. As the foundation of the economy, industry development with a green tone is one of the dimensions used to measure the level of green development in the Yangtze River Economic Belt.

Resource utilization, as one of the core elements of the green development of the Yangtze River Economic Belt, is an effective way to promote green development [38,39].

Environmental carrying capacity is one of the basic elements of green development [23,38,41]. In the context of rapid urbanization and increasing resource consumption intensity, environmental carrying capacity plays a crucial role in measuring the effectiveness of green development as a boundary that marks the scope of subsequent development efforts.

Environmental governance, as one of the core elements of the green development of the Yangtze River Economic Belt, is an important tool to guarantee green development [7,40,42].

Green life, as the embodiment of green development at the living level, is one way to materialize the effectiveness of green development in the Yangtze River Economic Belt [8,23]. The quality of green life is a symptom of the strength of people’s pursuit of a better life.

### 3.2. Evaluation Index System for Green Development Efficiency

Green development efficiency integrally reflects the coordinated development among the three systems of economy, society, and ecological environment, which not only considers the allocation efficiency of input and output factors in the process of economic and social development but also considers the pollution of the ecological environment. Based on the above green development system theory, input-output model, and the established research [4,7,44,45]; the index system of labor, fixed assets, energy consumption, water supply, and land use as input indicators; economic output, ecological environment, and social output as desired outputs; and wastewater emission, smoke and dust emission, exhaust gas emission, and air pollution as nonconsensual outputs are used to measure green development efficiency. Table 2 presents the evaluation index system for green development efficiency.

## 4. Materials and Methods

### 4.1. Study Area: Yangtze River Economic Belt

According to the official document “Guidance of the State Council on Promoting the Development of Yangtze River Economic Belt by Relying on the Golden Waterway” (http://www.gov.cn/zhengce/content/2014-09/25/content_9092.htm, accessed on 8 June 2022), the Yangtze River Economic Belt covers 11 provinces and cities, including Shanghai, Jiangsu, Zhejiang, Anhui, Jiangxi, Hubei, Hunan, Chongqing, Sichuan, Yunnan, and Guizhou, covering an area of approximately 2.05 million square km and spanning three regions in eastern, central, and western China. Among them, Shanghai, Jiangsu, and Zhejiang belong to the eastern region; Anhui, Jiangxi, Hubei, and Hunan belong to the central region; and Chongqing, Sichuan, Yunnan, and Guizhou belong to the western region [46].

**Figure 2:**
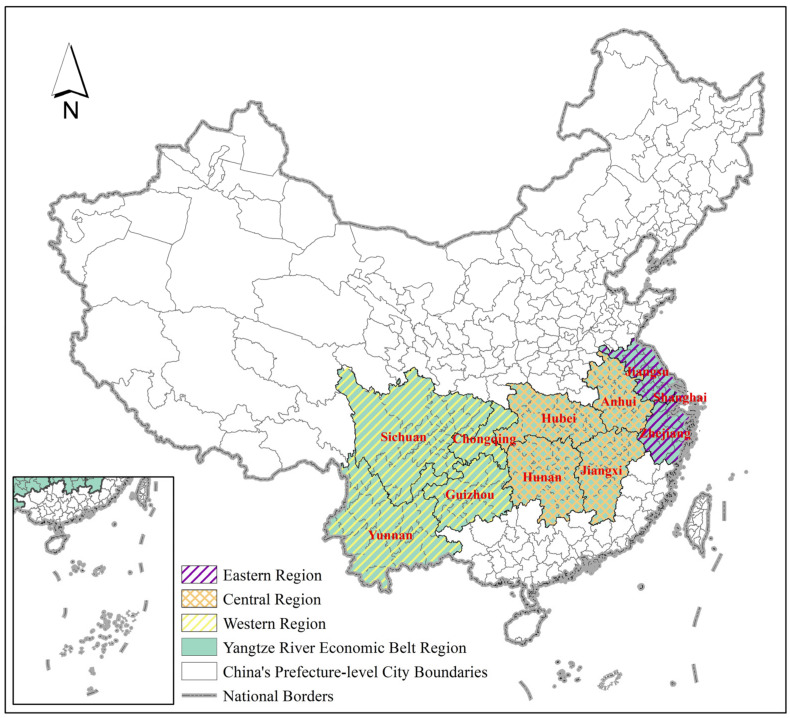
Study Area.

The geographical location of the study area is shown in Figure 2.

### 4.2. Methods

#### 4.2.1. Entropy Weight TOPSIS

The entropy weight TOPSIS method is a combination of the entropy weight and TOPSIS. The entropy weight was used to assign the weights of evaluation indicators, which was based on the degree of variability of the evaluation indicators, thus avoiding the influence of bias caused by human factors [5]. The order preference by similarity to ideal solution (TOPSIS) method, also known as the distance method of superiority and inferiority solution, is a multi-attribute decision-making method. The traditional TOPSIS method has the same weight for each evaluation index, which cannot reflect the difference in importance between different indices and has certain defects and limitations in application [6]. The improved entropy weight TOPSIS method can objectively reflect the relative importance of each evaluation index and is a comprehensive evaluation method with scientific analysis, validity, and accuracy [8]. Due to space limitations, the calculation steps were referred to in Li et al. [47].

#### 4.2.2. Super-EBM Model

The following three main methods for measuring green development efficiency are popular: data envelopment analysis (DEA), Solow residual method, and stochastic frontier analysis [28]. Among them, DEA is simple and easy to implement and is also used by most researchers. More importantly, the nonparametric directional distance function in DEA can consider both desirable and undesirable outputs [48]. Based on the theory proposed by Tone and Tsutsui, green development efficiency was measured based on the super-efficiency EBM (Epsilon-based measure) model built on the traditional DEA model [49], which is as follows:

(1) $$r^{*}=min\frac{\theta -\varepsilon^{-}\sum_{i=1}^{m}\frac{w_{i}^{-}s_{i}^{-}}{x_{i0}}}{\varphi +\varepsilon^{+}\left(\sum_{r=1}^{s}\frac{w_{r}^{+}s_{r}^{+}}{y_{r0}}+\sum_{p=1}^{q}\frac{w_{p}^{u-}s_{p}^{u-}}{u_{p0}}\right)}$$

(2) $$\begin{array}{c} s.t.\sum_{j=1}^{n}x_{ij}\lambda_{j}+s_{i}^{-}=\theta x_{i0}\\ \sum_{j=1}^{n}y_{rj}\lambda_{j}-s_{r}^{+}=\phi y_{r0}\\ \sum_{j=1}^{n}u_{pj}\lambda_{j}+s_{p}^{-}=\phi u_{p0} \end{array}$$

where $r^{*}$ is the optimal efficiency value satisfying $0\leq r^{*}\leq 1$. $x_{i0}$ is the input of $DMU_{0}$, $y_{r0}$ is the desired output, and $u_{p0}$ is the undesired output. $s_{i}^{-}$ denotes the input slack of $DMU_{0}$, and $s_{r}^{+}$ and $s_{p}^{-}$ are the desired and undesired output slack, respectively. $w_{i}^{-}$, $w_{r}^{+}$, and $w_{p}^{u-}$ denote the importance of each input, desired output, and undesired output, respectively. θ is the efficiency value in the radial condition. ε is the core parameter for the importance of the nonradial part, which satisfies 0≤ε≤1.

#### 4.2.3. Exploratory Spatial Data Analysis (ESDA)

Exploratory spatial data analysis refers to a collection of spatial data analysis methods and techniques that reveal spatial clustering, spatial anomalies, and spatial interactions among research objects through the visualization of spatial distribution patterns [50]. In this study, we utilized ArcGIS 10.2 and GeoDa to visualize spatiotemporal evolution and local spatial autocorrelation analysis (LISA). Then, the spatiotemporal patterns of green development performance and spatial autocorrelation were explored [51]. Among them, LISA was used to explore the local spatial variation pattern of the research object or the spatial anomalies that occur, which can be divided into the following four cases: high-high cluster, high-low cluster, low-high cluster, and low-low cluster. The formulation of LISA is specified as follows [52]:

(3) $$LISA_{i}=Z_{ij}\sum_{j}W_{ij}Z_{j}$$

where $Z_{i}$ and $Z_{j}$ denote the normalization of observations in region *i* and region *j*, respectively. $W_{ij}$ is the spatial weight, and alternatively, $\sum_{j}W_{ij}=1$.

#### 4.2.4. Geographic Detector (GD)

The GD method is widely used for the identification of the mechanisms of action of social, economic, and natural environmental factors because it is subject to fewer assumptions [43]. In addition, the use of GD can avoid the problem of multicollinearity; i.e., it can better avoid the problem of endogeneity in which the independent and dependent variables are mutually causal [53].

Factor detection is mainly used to detect the spatial heterogeneity of the explanatory variables and the extent to which influencing factor X explains the spatial heterogeneity of the explanatory variables, measured by the *q*-value. In this study, we mainly used factor detection and interaction detection. The expressions are as follows [54]:

(4) $$q=1-\frac{\sum_{h=1}^{L}N_{h}\sigma_{2}^{h}}{N\sigma^{2}}=1-\frac{SW}{SST}$$

(5) $$SSW=\overset{L}{\underset{h=1}{\sum }}N_{h}\sigma_{2}^{h}$$

(6) $$SST=N\sigma^{2}$$

where *h* takes values from 1 to *L*, indicating the stratification of the explanatory variable or influencing factor. $N_{h}$ and *N* denote the number of cells in the whole area of stratum *h*. $\sigma_{2}^{h}$ and $\sigma^{2}$ denote the variance of the explanatory variables in stratum *h* and the whole area, respectively. *SSW* and *SST* are the sum of the variance within the stratum and the total variance in the whole area, respectively. *q* takes values from [0, 1], and the larger the value is, the more obvious the spatial heterogeneity of the explanatory variables is. If stratification is generated by the influencing factor, the larger the value of *q* is, and the stronger the influencing factor that explains the explained variables. The influencing factor explains 100 × *q*% of the explained variables. When the *q*-value is 1, it indicates that the influencing factor completely controls the spatial heterogeneity of the explained variables. When the *q*-value is 0, there is no relationship between the influencing factor and the explained variables.

### 4.3. Data Sources

The statistical data in the evaluation index system were obtained from the China City Statistical Yearbook (2005–2018), the provincial and municipal statistical yearbooks (2005–2018), and the Statistical Bulletin of National Economic and Social Development of the corresponding cities. Missing data were complemented by a linear interpolation method.

In addition, with the support of GIS remote sensing data processing, the problem of accessibility of undesirable environmental outputs and other indicators not included in the statistical yearbooks was solved. The sources of remote sensing data are remote sensing monitoring data of Land-Use Current Conditions in China (LUCC), DMSP/OLS global nighttime lighting data, global PM_2.5_ inversion data, and MODND1 M monthly synthetic index of TERRA, a synthetic product of the International Scientific Data Mirror Center of the Computer Network Information Center of the Chinese Academy of Sciences.

## 5. Results

### 5.1. Spatiotemporal Characteristics of Green Development Performance

#### 5.1.1. Green Development Level

By averaging the green development level measurement results of 107 prefecture-level cities in the Yangtze River Economic Belt from 2004 to 2017, the ranking over 14 years was obtained. Shanghai, Chengdu, Chongqing, Hangzhou, Wuhan, Nanjing, Suzhou, Wuxi, Changsha, and Kunming, which are mostly provincial capitals or municipalities directly under the central government, have richer resources and better economic development than other cities. There were no eastern cities in the bottom ten of the ranking, 70% of which were western cities. In terms of variability, Shanghai was first in the green development level, with an index average of 0.843047, while Chengdu was second, with an index average of 0.405149, indicating that the green development level after second place showed a precipitous decline. In addition, the average value of the last ten cities was below 0.025, which was a large gap with the better cities. Most of the cities with high green development levels were eastern cities, and they were stable at a high level from 2004 to 2017, while most of the western and central cities continue to stay at a low level and had no obvious direction to improve. The above analysis showed that the green development of the Yangtze River Economic Belt had an obstacle-filled and long way to go.

**Figure 3:**
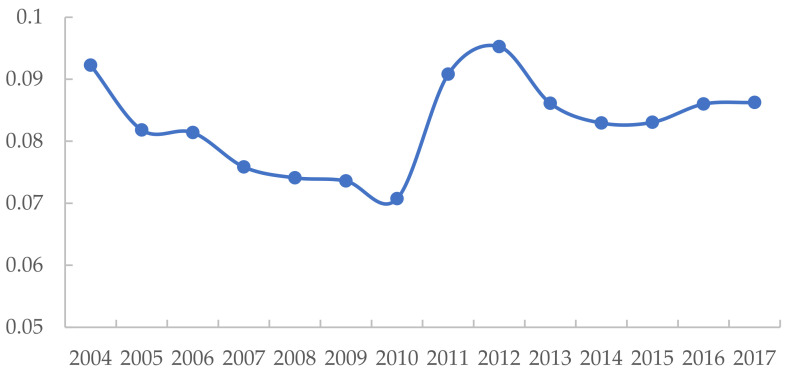
Average change trend of the green development level.

In addition, Figure 3 displays the evolution pattern of the average green development level, which shows an inverted N-shaped trend; i.e., it has three-stage evolution characteristics during the study period. In the first stage (2004–2009), international trade cooperation was gradually carried out after China’s accession to the World Trade Organization, which improved China’s backward production technology conditions but also caused ecological and environmental problems, such as increased environmental pollution and waste of resources, resulting in a downward trend in the green development level. In the second stage (2010–2012), the green development level rapidly improved, and the government adopted specific policy measures and set binding targets such as energy conservation and emission reduction, which had a significant effect on improving the green development level. In the Twelfth Five-Year Plan implemented in the third stage (2013–2017), the government paid more attention to structural and regional coordinated development. Therefore, the green development level gradually changed to a gentle increase after a small decline due to the initial adjustment.

**Figure 4:**
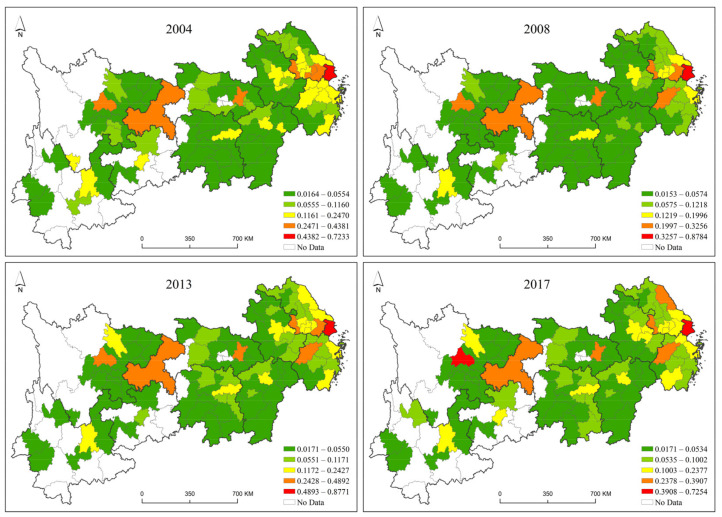
Spatial and Temporal Patterns of Green Development Level.

The above analysis based on the evolution trend of the time series ignores the feature exploration of the evolution trend of the green development level from a spatial perspective. Therefore, with the help of the Jenks natural breaks classification method in ArcGIS software, the green development level values from 2004 to 2017 were clustered separately by year and visualized, as shown in Figure 4, to display the spatial divergence.

In terms of the eastern region, the green development level had a leading role for the whole Yangtze River Economic Belt. Shanghai had outstanding advantages in the level of green development during the study period, which was matchless. Suzhou and Hangzhou, as developed cities in the eastern region, have a good level of economic development and an early start in environmental protection, so they have a good level of green development and an optimistic outlook. In addition, there were few cities in the eastern region with a low level of green development.

In terms of the central region, the three provincial capitals (Wuhan, Changsha, and Nanchang), as the core growth poles of green development in this region, were in the top three in terms of green development level. During the study period, the three provincial capitals had the advantages of strength, concentrated resources, and advanced technology, and their leading pattern has never changed. The reason could be that they signed the Agreement on Strategic Cooperation to Accelerate the Construction of the Midstream City Cluster of the Yangtze River in February 2012 and formally proposed building the Midstream City Cluster of the Yangtze River to lead the collaborative development of the central region. The green development level of other cities in the central region was low, which was related to improper environmental management, unreasonable industrial structure, and poor eco-environment. The status urgently needed to be adjusted, and the green development level also needed to be improved.

For the western region, the two growth poles of Chengdu-Chongqing and Kunming-Guiyang emerged significantly during the study period, and the clustering of these two poles formed a stable green development plateau in the western region. The remaining cities in the western region need to make efforts to transform and improve the green development level whether in terms of urban construction, industrial upgrading, or technological innovation.

**Figure 5:**
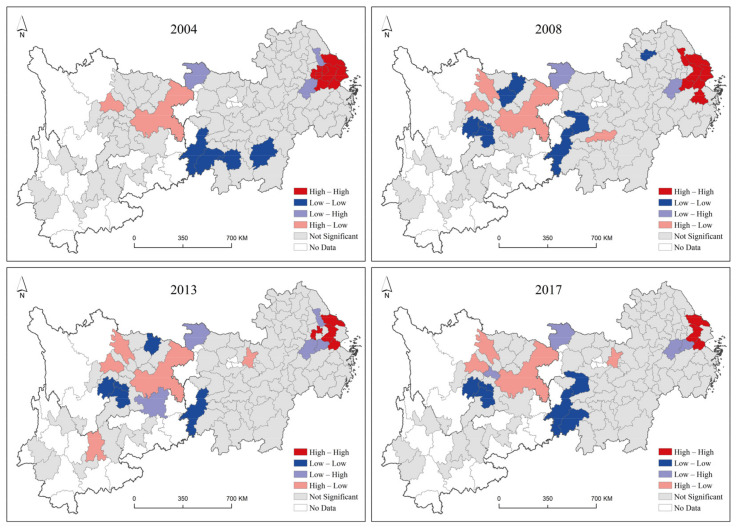
Local spatial autocorrelation clustering map of green development level.

Furthermore, using the spatial autocorrelation clustering method (LISA), the autocorrelation of the green development level of neighboring prefecture-level cities in the Yangtze River Economic Belt was identified and classified into the following four types of clusters: high-high cluster (H-H), where cities with high green development levels were surrounded by cities with high green development levels; high-low cluster (H-L), where cities with high green development levels were surrounded by cities with low green development levels; low-high cluster (L-H), where cities with low green development levels were surrounded by cities with high green development levels; and low-low cluster (L-L), where cities with low green development levels were surrounded by cities with low green development levels. The spatial clustering characteristics of the green development level in 2004, 2008, 2013, and 2017 were identified using GeoDa software, and the LISA clustering results that passed the 0.05 significance level test were obtained, as shown in Figure 5.

The results showed that the number of cities of the H-H, L-H, H-L, and L-L agglomeration types were basically stable. The H-H agglomeration type mainly appeared in the eastern region, and the number of this agglomeration type decreases slightly over time. For the H-L agglomeration type, the cities were mainly found in Chengdu, Chongqing, and Wuhan. For the L-H agglomeration type, the geographic locations of the cities were all around the H-H agglomeration type during the study period, and the overall changes in number and area were not significant. The L-L agglomeration type was basically scattered in the western and central regions, mainly appearing around Chongqing, and these cities were not driven by the radiation of Chongqing.

#### 5.1.2. Green Development Efficiency

**Figure 6:**
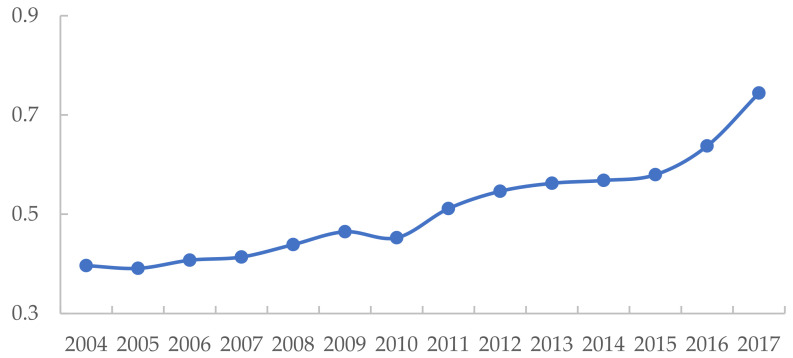
Average change trend of green development efficiency.

To portray the evolution of green development efficiency, the trend over the years is plotted as shown in Figure 6. It was easy to see that green development efficiency has fluctuated during the 14 years, but the overall trend was rising, with the highest mean value occurring in 2017. Green development efficiency declined slightly at the beginning of the study but had basically maintained an upward trend since 2006, probably because China had given more attention to energy conservation and environmental protection since 2006. The Eleventh Five-Year Plan and the Twelfth Five-Year Plan had set binding targets for energy conservation and emission reduction and enabled the adoption of relevant measures, such as eliminating backward production capacity and energy-saving actions for 1000 enterprises.

Although there were small fluctuations in green development efficiency, the overall trend was increasing year by year. The reason may be that the government has given more attention to the optimization of industrial structure, environmental pollution control, and coordinated regional development since the Eleventh Five-Year Plan. Therefore, economic strength has improved, and pollutant emissions have also been controlled accordingly. The green development efficiency increased from 0.396543 in 2004 to 0.744324 in 2017, an increase of 87.7%, with significant increases of 12.97% and 16.76% from 2010 to 2011 and 2016 to 2017, respectively.

**Figure 7:**
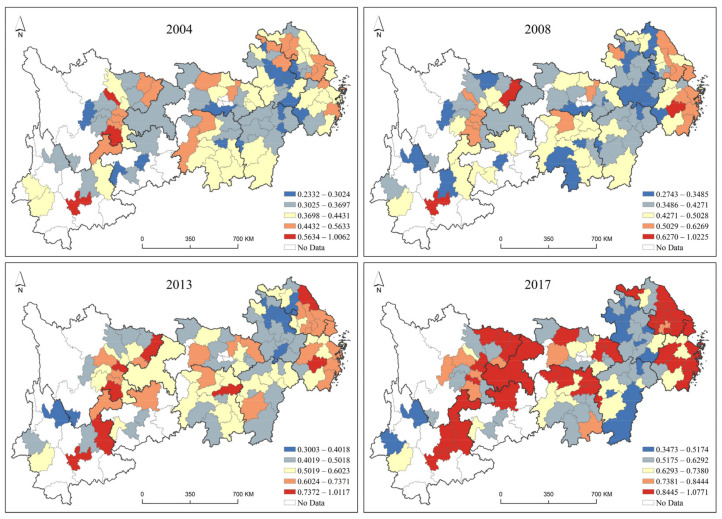
Spatial and Temporal Patterns of Green Development Efficiency.

The visualization is carried out with the Jenks natural breaks method in ArcGIS software, as shown in Figure 7.

From the results of the spatiotemporal pattern of green development efficiency, cities with high green development efficiency showed a development trend of gradually spreading from the eastern region to the central and western regions over time during the study period. The order from dark blue, light blue, yellow, and orange to red in the spatiotemporal pattern map corresponds to the low to high green development efficiency values, and the number of cities with red and orange in 2017 was significantly higher than that in 2004, indicating that the green development efficiency in the entire Yangtze River Economic Belt has significantly improved.

The green development efficiency showed a high spatial pattern in the eastern region and a low spatial pattern in the central and western regions. The Yangtze River Delta city cluster in the eastern region, as a developed region, is leading the Yangtze River Economic Belt in terms of both economic scale and city scale and is in the leading position in the eastern region in terms of green development efficiency. In addition, the middle reaches of the Yangtze River urban agglomeration perform better in the central region in terms of green development efficiency, and the Chengdu-Chongqing urban agglomeration, as a more developed area in the western region, performs better than other western cities.

Most of the low- and medium-efficiency cities are in the early stage of industrialization and are severely constrained by resource endowments. Their industrial structure is characterized by “two highs and one low”, which can easily lead to unsustainable development. The conflict between industrial structure and institutional mechanisms is the primary contradiction that makes it difficult for such cities to break through the bottleneck of green development efficiency improvement, and it is also the main reason why the green development efficiency of such cities has been at a low level.

**Figure 8:**
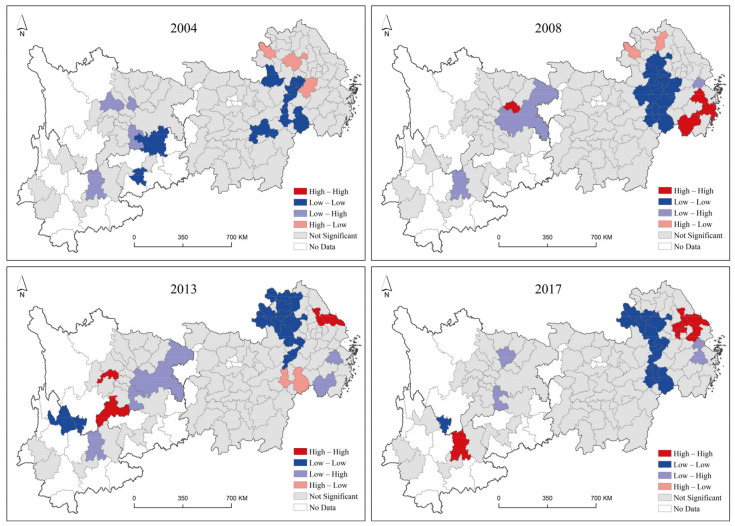
Local spatial autocorrelation clustering map of green development efficiency.

The spatial autocorrelation of the green development efficiency of cities was identified with the LISA method and classified into four types of clusters as follows: high-high cluster (H-H), where cities with high green development efficiency are surrounded by cities with high green development efficiency; high-low cluster (H-L), where cities with high green development efficiency are surrounded by cities with low green development efficiency; low-high cluster (L-H), where cities with low green development efficiency are surrounded by cities with high green development efficiency; and low-low cluster (L-L), where cities with low green development efficiency are surrounded by cities with low green development efficiency. Utilizing GeoDa software, the spatial clustering characteristics of green development efficiency in 2004, 2008, 2013, and 2017 were identified, and the LISA cluster map shown in Figure 8 was obtained. This result passed the 0.05 significance level test.

As shown in Figure 8, the number of cities in the H-H agglomeration increased significantly from 2004 to 2017, the change in the number of cities in the H-L agglomeration showed a downward then upward trend, and the change in the number of cities in the L-H agglomeration showed a downward then upward trend. The change in the number of cities in the L-L agglomeration showed a downward then upward trend, which is basically stable. For the H-H agglomeration type, the number of units was small at the beginning in the western region, and the geographical distribution of the agglomeration gradually shifted to the eastern region over time. For the H-L agglomeration type, the number of contained units was small, including zero in 2013, and its geographical distribution was mainly concentrated in the eastern region. For the L-H agglomeration type, cities were basically distributed in the western region from 2004 to 2013, mainly appeared around Chongqing and dispersed in all directions, and the number of cities in this agglomeration decreased significantly in 2017. The L-L agglomeration type mainly appeared in the middle and eastern regions, and the area centered on Hefei and Nanchang spread to the northern part of Anhui Province year by year. Although this agglomeration straddles the eastern region, it does not receive radiation from the eastern region, but its green development is significantly inhibited by the transfer of polluting industries from the eastern region. Therefore, it is necessary to strengthen the support for this region in terms of industrial optimization and upgrading and capital investment to prevent it from becoming a dark area for green development in the Yangtze River Economic Belt.

### 5.2. Analysis of Factors Influencing Green Development Performance

#### 5.2.1. Green Development Level

The green development of the Yangtze River Economic Belt is constrained by various social, economic, and environmental influences. The impact factors were treated as type variables using an equidistant discretization function, and then, the magnitudes of the factor forces in 2004, 2008, 2013, and 2017 were identified with the help of GD to reveal the impact mechanism.

The results of factor detection are shown in Table 3, which shows that the number of employees in scientific research, technical services, and geological exploration, value added of secondary industry, value added of tertiary industry, number of urban public toilets, per capita retail sales of social consumer goods, and total public library book collection per capita were all high-impact factors in 2004, 2008, 2013, and 2017. The results indicated that the important influencing factors of the green development level were mainly concentrated in the layer of economic development, industrial development criteria, and green life. With the improvement in the economic level, people’s demand for material culture and the quality of development and environmental benefits have gradually increased. Therefore, we should actively take measures to improve production technology and build sewage-cleaning facilities so that economic development and industrial development can be made sustainable, and people’s welfare can be improved, therefore improving the green development level.

The evolution of the explanatory strength of the factors (ranking changes) shows that most rankings of the factors were stable during the study period. It is worth noting that the value added of the tertiary industry was ranked in the top two in the study period, which reflects that green development mainly relies on the improvement of tertiary industry. Therefore, to improve the green development level, emphasis should be placed on the rationalization of industrial structure and the cleanliness of production technology.

#### 5.2.2. Green Development Efficiency

The improvement of green development efficiency and the evolution of its spatial pattern are closely related to various economic and social factors. The influencing factors are interactively coupled and influence each other, which together lay the formation of the spatial pattern of green development efficiency in the municipalities of the Yangtze River Economic Belt. Referring to the established literature [4,48,55,56,57,58], eight influencing factors, including economic development, industrial structure, population density, science and technology expenditure, education level, government support, energy consumption, and opening up, were selected, and detailed descriptive statistical information and variable symbols are shown in Table 4. The following multiple regression econometric model was constructed:

(7) $$efficiency_{it}=\beta_{0}+\beta_{1}eco_{it}+\beta_{2}ind_{it}+\beta_{3}pop_{it}+\beta_{4}tec_{it}+\beta_{5}edu_{it}+\beta_{6}gov_{it}\;+\beta_{7}energy_{it}+\beta_{8}open_{it}+u_{i}+\varepsilon_{it}$$

where $efficiency_{it}$ denotes the green development efficiency of city *i* in year *t*. The specific meanings of the influencing factors are detailed in Table 4; $\beta_{0}$ is the intercept term, and $\varepsilon_{it}$ is the random disturbance term.

(1) Economic development (*eco*): The higher the economic development level is, the higher the material base and technology level. Simultaneously, the same output requires less resource input, and the efficiency of green development can be increased [55]. The expected sign was positive.

(2) Industrial structure (*ind*): It has been demonstrated that industrial structure optimization can significantly improve green development efficiency [56]. The share of secondary industry in GDP was used as a proxy; therefore, the expected sign was negative.

(3) Population density (*pop*): Population density reflects the population carrying capacity of a city; the higher the population density is, the lower the ecological carrying capacity, and the more vulnerable the ecosystem is to damage, thus inhibiting green development [4]. The expected sign was negative.

(4) Science and technology expenditure (*tec*): The higher the level of technological innovation is, the easier it is to achieve green technological improvements, which in turn have a positive impact on the efficiency of green development [57]. Therefore, the expected sign was positive.

(5) Education level (*edu*): Tong et al. found that education level is an important factor in enhancing green development efficiency [48]. The expected sign was positive.

(6) Government support (*gov*): The government’s macroregulation may have a positive effect on the improvement of green development efficiency; however, the unreasonable behavior of local governments may also cause environmental pollution [48]. Therefore, the expected sign was uncertain.

(7) Energy consumption (*energy*): As a major coal country, energy consumption in China usually means consuming more coal and causing environmental pollution [58], which in turn is not conducive to green development efficiency. Therefore, the expected sign was negative.

(8) Opening up (*open*): The opening up may have two effects on the city, namely a “pollution paradise” or a “pollution halo” [55], so the expected sign was uncertain.

The models were estimated with Stata 15.0 software. In Table 5, model (1) reports the results of the panel-mixed least squares estimation, model (2) reports the results of the fixed effects estimation, model (3) reports the results of the two-way fixed-effects model estimation, and model (4) reports the results of the panel-corrected standard deviation estimation using Prais–Winsten regression.

First, the panel ordinary least squares (OLS) with fixed-effects model was used for estimation, and the results are reported in columns of model (1) and model (2), with the panel-setting F test result of 24.6 and significant at the 1% level; i.e., the fixed effects estimation is better than the mixed estimation. The Breush–Pagan LM test result was significant at the 1% level of 2422.37, indicating that random effects estimation was better than mixed estimation. The Hausman test result was 115.68 and significant at the 1% level, which indicated that the fixed effects model was better than the random effects model; in other words, the original hypothesis of no difference between fixed effects and random effects regression coefficients was significantly rejected.

To examine the issue of unobservable intermunicipal unit heterogeneity and time effects, year dummy variables were included, regressions were estimated with a two-way fixed model, and the results are shown in the column of model (3). From the regression results of model (3), all the influencing factors positively and significantly affect green development efficiency except for science and technology expenditure, education level, and opening up. Since this section uses panel data to investigate the factors influencing green development efficiency, we had to consider the characteristics of cross-sectional data and time series data of the panel data itself. The modified Wald test results significantly reject the original hypothesis at the 1% level; i.e., there is intergroup heteroskedasticity in the panel data model. The Wooldridge test result was 58.14, which significantly rejected the original hypothesis; i.e., the panel data model was determined to have intragroup autocorrelation. The Pesaran test statistic was 45.288, which was significant at the 1% level, and the Frees test statistic was 11.735, significant at the 1% level. Both of these cross-sectional correlation tests rejected the original hypothesis, indicating the existence of cross-sectional autocorrelation in the panel data model. Therefore, this section regresses the model using the Prais–Winsten method of PCSE (panel-corrected standard errors), and the results are reported in column 4 (model (4)).

Based on the analysis of the above test results, this section finally takes the regression results of the PCSE model as the benchmark. From the results shown in the column of model (4), it can be seen that when the model was applied to a more stringent statistical test, the regression coefficients of industrial structure, population density, science and technology expenditure, education level, and government support are not significant, which indicates that there is a weak effect on green development efficiency, among which the regression coefficient of government support was positive. Therefore, policy makers need to increase support to promote comprehensive green transformation. Economic development has a significant positive effect, with green development efficiency rising by 0.2803% for every 1% increase in the economic development level. As the level of economic development continues to increase, the efficiency of resource utilization and pollution control capacity also increase, and therefore, green development efficiency will be improved. It follows that solid economic strength can provide a material basis for the sustainable promotion of green development under the premise of safeguarding environmental quality. Energy consumption has a significant negative impact on green development efficiency. For every 1% decrease in energy consumption per unit of GDP, green development efficiency increases by 0.1401%; therefore, only by improving energy utilization and vigorously promoting the use of clean energy can green development efficiency be improved. The finding of a significant inhibitory effect of external openings on green development efficiency demonstrates the existence of the pollution paradise hypothesis [59], which indicates that most of the foreign-funded enterprises introduced by the municipalities in the Yangtze River Economic Belt are polluting and have caused irreversible damage to the ecological environment.

## 6. Conclusions and Discussions

### 6.1. Conclusions

(1) The trend of the green development level in the Yangtze River Economic Belt from 2004 to 2017 had an inverted “N” shape, with obvious three-stage evolutionary characteristics. The green development efficiency has steadily increased from 0.396543 to 0.744324, with an overall increase of 87.7%.

(2) In terms of the spatial and temporal evolution pattern of the green development level, two core growth poles, the Yangtze River Delta region and Chengdu-Chongqing urban agglomeration, were formed during the study period, and the H-H cluster mainly appeared in the eastern region, while the L-L cluster was mainly distributed in the western region. For green development efficiency, the spatial pattern of high in the eastern region and low in the central and western regions is similar to the spatial pattern of the green development level, but the L-L cluster is mostly distributed around the H-H cluster.

(3) The top five influencing factors of the green development level in 2004 are X10 > X9 > X29 > X34 > X2 in order of strength, X9 > X10 > X29 > X2 > X6 in 2008, X10 > X2 > X29 > X9 > X6 in 2013, and X10 > X2 > X9 > X29 > X18 in 2017, which reflects that industrial structure and people’s welfare are still important influencing factors of the green development level in the Yangtze River Economic Belt, and the impact of industrial structure optimization was more significant.

(4) According to the regression results of the PCSE model, the improvement in the green development efficiency of the Yangtze River Economic Belt was mainly driven by economic development, and the inhibiting effect of energy consumption was significant. In addition, the effect of opening up has not yet changed from a “pollution paradise” to a “pollution halo”.

### 6.2. Discussions

Based on the empirical results, it is easy to conclude that it is not scientific to explore how to promote green development in the Yangtze River Economic Belt only from a single perspective of level or efficiency, which may be a new advancement by breaking the conventional thinking of the established literature in the field of green development focusing only on level or efficiency. First, the trends of the green development level and green development efficiency are not consistent. The change in the green development level is more likely to be influenced by policy implementation, while green development efficiency is steadily improving with economic development and clean technology progress. Therefore, to comprehensively promote green development, attention should be given to guaranteeing the steady improvement of the green development level when relevant policies are formulated. Second, compared with the green development level, there are more L-L clusters in the spatial and temporal pattern of green development efficiency, and most of them appear in the near central region adjacent to the Yangtze River Delta, which indicates that the phenomenon of “central collapse” is more significant in the spatial and temporal patterns of green development efficiency. This means that the central region needs to pay more attention to the improvement of input-output efficiency instead of focusing on the elevated level of green development and ignoring efficiency.

Therefore, based on the conclusions and the above analysis, the following policy insights are presented:

(1) Giving a leading role to the eastern region: From the spatial and temporal evolution of green development performance, it can be seen that the green development level and green development efficiency are both “high in the east and low in the west”, and the higher level of green development performance is concentrated in the east. Therefore, to reduce the differences in green development performance between cities, the radiation-driven role of the eastern region should be given full play, and the role of helping the central and western regions in terms of technological innovation, industrial structure upgrading, and capital investment should be increased to reduce regional differences and promote green development in the whole region.

(2) Formulating appropriate green development policies: First, the advanced experience of developed regions or countries can be learned. For example, the United States promotes green development by investing in clean energy [60], the EU has established green industries, and Japan has implemented policies such as a low-carbon society, material recycling society, and harmony with nature [61]. Second, from the results of the analysis of the influencing factors, the relevant policy makers can promote green development by promoting industrial upgrading, introducing clean technology, increasing environmental protection, and abandoning the crude economic development model.

### 6.3. Limitations and Future Directions

This study provides a comprehensive assessment of the green development performance of the Yangtze River Economic Belt from two aspects, namely level and efficiency, which has certain practical significance for promoting green development. However, there were still limitations. (1) This study examines green development performance at the prefecture-level city scale, which can be further explored at the county level based on the administrative division of China. (2) Due to the availability of data at the prefectural level, the statistical indicators used for green development performance assessment are limited, and further consideration can be given to include remote sensing indicators such as NO_2_, CO_2_, and the NDVI to enrich the evaluation system. (3) The Yangtze River Economic Belt was taken as the study area, which has a certain importance for promoting the overall green transformation in China, and subsequent studies with other regions as the study area are also necessary. (4) The study period in this paper has not yet covered the time period after the emergence of COVID-19, and further research is needed on the impact of COVID-19 on green development.

## Figures and Tables

**Table 1:** Evaluation Index System for Green Development Level.

Target Layer	Guideline Layer	Indicator Layer	Unit	Attributes
Green Development Level	Growth Quality	GDP per capita growth rate	%	+
		Number of employees in scientific research, technical services, and geological exploration	Million people	+
		Per capita amount of actual use of foreign capital in the year	USD/person	+
		Average profit of industrial enterprises above the scale	Million CNY/person	+
		Fixed asset investment per capita	CNY/person	+
		Per capita disposable income of urban residents	CNY	+
		The proportion of science expenditure to GDP in fiscal expenditure	%	+
	Industry Development	Value added of primary industry	Billion CNY	+
		Value added of secondary industry	Billion CNY	+
		Value added of tertiary industry	Billion CNY	+
		Value added of the tertiary industry as a proportion of GDP	%	+
		Growth rate of tertiary industry	%	+
		The proportion of tertiary industry employees	%	+
	Resource Utilization	Reduction rate of urban construction land area per unit GDP	%	+
		Reduction rate of total urban water supply per unit GDP	%	+
		Energy consumption per unit GDP		−
		Arable land retention per capita	Hectares per 10,000 people	+
	Environmental Carrying Capacity	Urban population density	People/km^2^	−
		Emission of industrial wastewater per unit land area	Million tons/square km	−
		Emission of industrial soot per unit land area	Ton/km^2^	−
		Industrial sulfur dioxide emissions per unit land area	Ton/km^2^	−
		Amount of fertilizer application per unit land area for agriculture	Ton/km^2^	−
		Industrial sulfur dioxide removal per unit land area	Ton/km^2^	−
	Environmental Governance	Urban domestic sewage treatment rate	%	+
		Harmless treatment rate of domestic waste	%	+
		PM_2.5_	Index	−
		Green coverage rate of built-up areas	%	+
	Green Life	Park green area per capita	Square m/person	+
		Number of urban public toilets	Square m	+
		Urban road area per capita	Square m	+
		Density of drainage pipes in built-up areas	km/km^2^	+
		Urban water penetration rate	%	+
		Urban gas penetration rate	%	+
		Per capita retail sales of social consumer goods	CNY/person	+
		Total public library book collection per capita	Thousands of books, pieces/10,000 people	+
		Number of hospital and health center beds per capita	Sheets/10,000 people	+

**Table 2:** Evaluation Index System for Green Development Efficiency.

Target Layer	Guideline Layer		Indicator Layer	Unit
Green Development Efficiency	Inputs	Workforce	Number of employees	Person
		Fixed assets	Investment in fixed assets	CNY
		Energy consumption	Energy consumption	Index
		Water supply	Total urban water supply	%
		Land use	Arable land area	Hectares
			Urban construction land area	Square km
	Desired outputs	Economic output	GDP	Million CNY
			Value added of tertiary industry	Billion CNY
		Ecological environment	Park green space area	m^2^
		Social output	Total retail sales of social consumer goods	CNY
			Public library book collection	Thousands of books
	Undesired outputs	Wastewater emissions	Industrial wastewater emissions	Million tons
		Fume and dust emissions	Industrial smoke and dust emissions	Ton
		Exhaust emissions	Industrial sulfur dioxide emissions	Ton
		Air pollution	PM_2.5_	μg/m^3^

**Table 3:** Detection results for green development level factors.

Detection Factors	2004			2008			2013			2017		
	qv	sig	Rank	qv	sig	Rank	qv	sig	Rank	qv	sig	Rank
GDP per capita growth rate (X1)	0.0998	0.0457	30	0.1699	0.0008	23	0.0835	0.0424	28	0.0810	0.0949	25
Number of employees in scientific research, technical services, and geological exploration (X2)	0.6492	0.0000	5	0.8121	0.0000	4	0.8109	0.0000	2	0.8648	0.0000	2
Per capita amount of actual use of foreign capital in the year (X3)	0.5693	0.0000	7	0.4958	0.0000	11	0.5896	0.0000	7	0.4889	0.0000	7
Average profit of industrial enterprises above the scale (X4)	0.2365	0.0001	20	0.1062	0.0358	27	0.0537	0.2979	34	0.1505	0.0023	16
Fixed asset investment per capita (X5)	0.4496	0.0000	10	0.4284	0.0000	13	0.2029	0.0006	17	0.1381	0.0097	18
Per capita disposable income of urban residents (X6)	0.3803	0.0000	14	0.7679	0.0000	5	0.6421	0.0000	5	0.5241	0.0000	6
The proportion of science expenditure to GDP in fiscal expenditure (X7)	0.3414	0.0000	15	0.2963	0.0278	16	0.2788	0.0296	14	0.2354	0.0179	13
Value added of primary industry (X8)	0.1184	0.0287	27	0.0961	0.2588	28	0.1256	0.0219	21	0.2031	0.0196	14
Value added of secondary industry (X9)	0.7711	0.0000	2	0.9240	0.0000	1	0.7669	0.0000	4	0.7577	0.0000	3
Value added of tertiary industry (X10)	0.8269	0.0000	1	0.9119	0.0000	2	0.9169	0.0000	1	0.8678	0.0000	1
Value added of the tertiary industry as a proportion of GDP (X11)	0.2032	0.0022	22	0.2929	0.0000	17	0.3219	0.0003	13	0.2739	0.0007	12
Growth rate of tertiary industry (X12)	0.0370	0.4481	36	0.0304	0.5471	36	0.0691	0.0774	31	0.1113	0.0217	20
The proportion of tertiary industry employees (X13)	0.1407	0.0109	25	0.1104	0.0049	26	0.0521	0.1812	35	0.0386	0.2756	34
Reduction rate of urban construction land area per unit GDP (X14)	0.2570	0.0077	19	0.0698	0.3179	32	0.0641	0.1775	33	0.0962	0.0244	22
Reduction rate of total urban water supply per unit GDP (X15)	0.0430	0.2315	34	0.1400	0.0087	24	0.0746	0.0263	29	0.0255	0.6303	36
Energy consumption per unit GDP (X16)	0.3931	0.0000	13	0.1314	0.0125	25	0.1138	0.0114	23	0.1062	0.0159	21
Arable land retention per capita (X17)	0.2763	0.0030	17	0.4392	0.0002	12	0.2281	0.0002	16	0.3209	0.0001	11
Urban population density (X18)	0.4273	0.0000	11	0.6828	0.0000	6	0.5055	0.0000	9	0.5339	0.0000	5
Emission of industrial wastewater per unit land area (X19)	0.4791	0.0000	9	0.3828	0.0004	14	0.4448	0.0008	11	0.4397	0.0000	10
Emission of industrial soot per unit land area (X20)	0.2690	0.0002	18	0.2022	0.0022	20	0.1703	0.0107	19	0.1400	0.0089	17
Industrial sulfur dioxide emissions per unit land area (X21)	0.5353	0.0000	8	0.6704	0.0000	8	0.2618	0.0824	15	0.0646	0.1738	30
Amount of fertilizer application per unit land area for agriculture (X22)	0.0822	0.0429	31	0.0727	0.1292	31	0.0985	0.0215	27	0.1258	0.0164	19
Industrial sulfur dioxide removal per unit land area (X23)	0.3165	0.0001	16	0.2340	0.0007	18	0.1815	0.0032	18	0.0715	0.1063	28
Urban domestic sewage treatment rate (X24)	0.0645	0.1745	33	0.2051	0.0002	19	0.1029	0.0415	26	0.0535	0.0698	33
Harmless treatment rate of domestic waste (X25)	0.2076	0.0000	21	0.1918	0.0001	21	0.3595	0.0000	12	0.0694	0.1826	29
PM_2.5_ concentration (X26)	0.0405	0.0823	35	0.0453	0.2109	35	0.0653	0.1518	32	0.0573	0.2253	32
Greening coverage rate of built-up areas (X27)	0.1921	0.0008	23	0.0826	0.0890	29	0.1142	0.0116	22	0.0346	0.3238	35
Park green area per capita (X28)	0.1187	0.0227	26	0.0481	0.1878	34	0.0293	0.6637	36	0.0719	0.1881	27
Number of urban public toilets (X29)	0.7080	0.0000	3	0.8626	0.0000	3	0.7865	0.0000	3	0.7133	0.0000	4
Urban road area per capita (X30)	0.1013	0.0521	29	0.0605	0.2437	33	0.0742	0.3035	30	0.0799	0.3799	26
Density of drainage pipes in built-up areas (X31)	0.0780	0.0967	32	0.0764	0.0236	30	0.1304	0.0482	20	0.0841	0.0825	24
Urban water penetration rate (X32)	0.1084	0.0548	28	0.1760	0.0010	22	0.1050	0.0123	25	0.0865	0.0634	23
City gas penetration rate (X33)	0.1605	0.0137	24	0.6805	0.0000	7	0.5929	0.0000	6	0.0629	0.1816	31
Per capita retail sales of social consumer goods (X34)	0.6563	0.0000	4	0.5395	0.0000	10	0.4927	0.0000	10	0.4683	0.0000	8
Total public library book collection per capita (X35)	0.5882	0.0000	6	0.5815	0.0000	9	0.5774	0.0000	8	0.4464	0.0006	9
Number of hospital and health center beds per capita (X36)	0.3956	0.0000	12	0.3191	0.0000	15	0.1054	0.1433	24	0.1757	0.0322	15

**Table 4:** Summary of Variable Information and Descriptive Statistics.

Influencing Factors	Proxy Variables	Unit	Mean	Max	Min	S.D.	Symbol
Economic Development	GDP per capita	CNY	10.15	12.00	7.95	0.80	*eco*
Industrial Structure	Share of secondary industry in GDP	%	3.89	4.48	2.93	0.22	*ind*
Population Density	Urban population density	People/km^2^	6.02	7.74	3.97	0.61	*pop*
Science and Technology Expenditure	Share of fiscal expenditure on science in GDP	%	−6.63	−3.94	−17.40	1.19	*tec*
Education Level	Share of educated population above high school	%	−1.83	−0.76	−2.88	0.43	*edu*
Government Support	Fiscal expenditure as a proportion of GDP	%	−1.91	7.47	−6.91	0.56	*gov*
Energy Consumption	Energy consumption per unit GDP	-	4.25	5.87	2.76	0.48	*energy*
Opening up	The proportion of actual foreign investment used in the year to GDP	%	−7.79	−3.23	−14.66	1.93	*open*

**Table 5:** Model estimation results.

Variables	Model (1)	Model (2)	Model (3)	Model (4)
*eco*	0.3427 ***	0.3065 ***	0.1161 ***	0.2803 ***
*ind*	−0.1593 ***	−0.1821 ***	0.1681 ***	−0.0545
*pop*	−0.0564 **	0.1807 ***	0.2455 ***	−0.0273
*tec*	0.01261 ***	−0.0154 ***	−0.0087	0.00001
*edu*	−0.0312	0.0777 ***	0.0845	−0.1294
*gov*	−0.0046	−0.0361 ***	0.0336 ***	0.0149
*energy*	−0.2077 ***	−0.2434 ***	0.2670 ***	−0.1401 ***
*open*	−0.0236 ***	−0.0137 **	−0.0030	−0.0166 **
constant	−2.695 ***	−3.315 ***	1.6931 ***	−2.9090 ***
Panel-setting F test		24.6 ***		
Hausman test		115.68 ***		
Breush–Pagan LM test		2422.37 ***		
Modified Wald test				2576.14 ***
Wooldridge test				58.14 ***
Pesaran test				45.288 ***
Frees test				11.735 ***
R^2^	0.6040	0.6093	0.6657	0.5569
Observations	1498	1498	1498	1498

Note: ***: *p* < 0.01, **: *p* < 0.05, *: *p* < 0.1.

## Data Availability

Not applicable.
